# M1 Microglia Induced Neuronal Injury on Ischemic Stroke via Mitochondrial Crosstalk between Microglia and Neurons

**DOI:** 10.1155/2022/4335272

**Published:** 2022-11-28

**Authors:** Wei Liu, Zitong Qi, Wanmeng Li, Jia Liang, Liang Zhao, Yijie Shi

**Affiliations:** ^1^School of Pharmacy, Jinzhou Medical University, Jinzhou 121000, China; ^2^Life Science Institution, Jinzhou Medical University, Jinzhou 121000, China

## Abstract

Among the middle-aged and senile populations, ischemic stroke (IS) is a frequently occurring acute condition of the cerebrovascular system. Traditionally, it is recognized that when stroke occurs, microglia are activated into M1 phenotype and release cytotoxic cytokines, reactive oxygen species, proteases, and other factors, thus exacerbating the injury by further destroying or killing nearby neurons. In the latest research, the crucial role of the intercellular mitochondrial crosstalk on the stroke management has been demonstrated. Therefore, we tried to clarify mitochondrial crosstalk between microglia and neurons, and evaluated M1 microglial mitochondria-mediated neurological performance in transient middle cerebral artery occlusion (tMCAO) rats. We found that when microglia was activated into the proinflammatory M1 type after stroke, mitochondrial fission process was accelerated, and damaged mitochondria were released, further transferred to neurons and fused with neuronal mitochondria. As a result, the function of neuronal mitochondria was damaged by decreasing adenosine triphosphate (ATP), mitochondria membrane potential, and increasing excessive reactive oxygen species (ROS), thus inducing mitochondria-mediated neuronal death and finally aggravating ischemia injury. Taken together, it provides a novel neuroglial crosstalk mechanism at the mitochondrial level.

## 1. Introduction

Ischemic stroke (IS), as a neurological deficit syndrome caused by acute vascular or blood abnormalities [1], features high prevalence and mortality, as well as high recurrence and disability rates. One of the distinct traits of ischemic stroke has been regarded as mitochondrial disorder, which is a contributor to the pathology of IS [2]. Mitochondria are the major places for aerobic respiration and the energy factory within cells, which maintain cell homeostasis through calcium signal and cell metabolism [3]. Upon occlusion of the artery, depolarization of mitochondrial membrane commences in the ischemic brain, causing the adenosine triphosphate (ATP) consumption, unfolded protein response (UPR), excessive reactive oxygen species (ROS) production, and PTEN-induced putative kinase 1 (PINK1) generation [4]. As a result, it will trigger ischemic cascade reactions, including the 1-methyl-4-phenyl-1,2,3,6-tetrahydropyridine (MPTP) opening, caspase 3 initiation, and the cytochrome c secretion, so that the apoptotic execution occurs. In the latest research, it is found that mitochondrial crosstalk between cells exerts a crucial effect on the stroke management [5, 6]. In addition, extracellular mitochondria can be transferred to damaged neurons for aggravating or reducing the damage of neurons [7].

**Figure 1:**
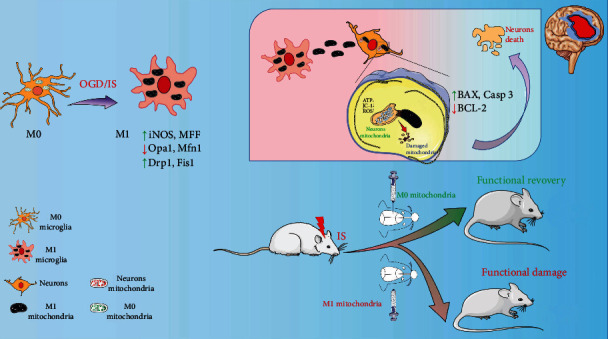
The major assumption of the present study. When IS occurred, more unhealthy mitochondria were secreted from M1 microglia and transferred into neurons for triggering mitochondria-mediated damage of neurons, thus aggravating ischemia-induced injury by enlarging infarct area and lowering neurological performance in transient middle cerebral artery occlusion (tMCAO) rats.

Microglia as the key neuroinflammatory mediator is capable of eliciting or regulating a broad spectrum of cellular responses [8]. IS induces M1 phenotype activation of microglia and enhances the secretion of proinflammatory factors such as interleukin-1 beta (IL-1*β*), inducible nitric oxide synthase (iNOS), and tumor necrosis factor alpha (TNF-*α*), thus inducing a cytokine storm of proinflammatory factors and accelerating neuronal injury [9]. Most previous research is focused on microglia-mediated inflammatory regulation. However, dysfunction of M1 microglial mitochondria, which is involved in diverse molecular and cellular functions, is not paid more attention for treating stroke in the clinical settings. In our study, as shown in Figure 1, it was found that when microglia were activated into M1 phenotype after ischemic stroke, the dynamic balance of mitochondria was destroyed as evidenced by the activation of mitochondrial fission and prohibition of mitochondrial fusion. As a result, marked pathological fragmentation of mitochondria was produced, and mitochondrial function was significantly disturbed by decreased ATP and increased ROS levels. Finally, damaged microglial mitochondria were further released, subsequently entered adjacent neurons, and induced fusion with neuronal mitochondria, thus exacerbating neuronal mitochondrial dysfunction, amplifying neuronal damage, and worsening neurological outcomes. In conclusion, activated microglia (M1) released damaged mitochondrial fragmentation out of cells after ischemia, ensuring the transmission of injured microglial mitochondria to neurons, triggering fusion with neuronal mitochondria, and inducing mitochondria-mediated neuronal damage and death.

## 2. Materials and Methods

### 2.1. Materials and Animals

Rabbit antibodies, such as TOM20 (WL0706), BAX (WL01637), BCL-2 (WL01556), cytochrome c (WL02410), caspase 3 (WL04004), iNOS (WL0992a), VDAC (WL02790), IL-2 (WL03259), TNF-*α* (WL01581), IL-1*β* (WLH3903), and *β*-actin (WL01372), were procured from Wanleibio in Shenyang, China, while rabbit anti-MFF (AF2365), anti-Mid49 (DF12044), anti-Mid51 (DF12019), anti-Opa1 (DF8587), and anti-Mfn1 (DF7543) were obtained from AFFINITY, Co., Ltd. This study acquired goat anti-rabbit IgG/HRP (PAB21463HRP-1000) secondary antibody (1 : 10000) from EarthOx (Millbrae, USA). The reagents and chemicals were all products of Sigma (St. Louis, USA). We procured SH-SY5Y cells from the Type Culture Collection of Chinese Academy of Sciences (Shanghai, People's Republic of China) and cultured them using DMEM/F-12 involving fetal bovine serum (FBS, 10%) and PS (penicillin–streptomycin, 1%). Meanwhile, BV2 microglial cells, which were purchased from the iCell Bioscience Inc., Shanghai, were cultured using DMEM (HyClone, UT, USA) involving FBS (10%; Gibco, CA, USA) and PS (1%). Male SD (Sprague–Dawley) rats, whose weights ranged from 250 to 280 g, were offered by Jinzhou Medical University. The present experimental protocol conformed to the National Guidelines for Animal Protection and gained approval from foregoing university's Institutional Animal Care and Use Committee.

### 2.2. Middle Cerebral Artery Occlusion/Reperfusion and In Vivo Treatments

Focal cerebral ischemia was induced by transient occlusion of the middle cerebral artery (tMCAO) using intraluminal filament technique [10]. Briefly, rats were anaesthetized, and the whole operation was conducted on a thermostatic blanket which was used to maintain the rectal temperature at about 37 ± 0.5°C. The one side common carotid artery (CCA), external carotid artery (ECA), and internal carotid artery (ICA) were exposed. Ligation of the CCA was performed with surgical nylon monofilament near the distal end of the CCA, and ligation of the ECA was conducted at two positions at the end of ECA and near ICA and ECA bifurcations. Then, between the two ECA ligatures, we made a small incision. Nylon filament about certain lengths was softly intercalated into the ICA through the ECA stump and pushed into the anterior cerebral artery until slight resistance was felt which was approximately the location of the mark place. After 2 h of ischemia, the filament was drawn out followed by applying appropriate drug to the surgical areas to prevent infection. After reperfusion, PBS, the released mitochondria collected from the culture medium of M0 microglia (Mito/M0-BV2) and M1 microglia (Mito/M1-BV2) were infused into the cerebral cortex [2].

### 2.3. Zea-Longa Score

The Zea-Longa test of tMCAO rats (*n* = 5) was performed 6 hours after reperfusion to assess neurobehavioral deficits, as previously described [11], and the Zea-Longa 5-point scoring standard included: 0 points: behavior is completely normal, without any symptoms of neurological deficit; 1 point: mild neurological deficit, dysfunction in stretching the left forelimb; 2 points: moderate neurological deficit, rats cannot go straight and walk forward because the body continues to turn to the side; 3 points: severe neurological deficit, rats cannot stand and fall to the left when standing; 4 points: rats cannot walk spontaneously, loss of consciousness; and 5 points: rats die.

### 2.4. Ludmila Belayev Score

The Ludmila Belayev test of tMCAO rats treated with different administrations (*n* = 5) was performed 6 hours after reperfusion to assess neurobehavioral deficits [12], and the Ludmila Belayev 12 scoring standard included lift suspension (2 points)—0 point: no obvious loss of nerve function; 1 point: mild flexion of the contralateral limb of infarction; and 2 points: obvious flexion of the contralateral limb of infarction. Limb placement was divided into visual subtest (front, side) and tactile subtest (front, side) (8 points)—0 point: the animal limb placement reaction was normal; 1 point: response delay but not more than 2 s; and 2 points: reaction delay and >2 s—and ontoception subtest (2 points)—0: as powerful as contralateral limb; 1: slightly more powerful than contralateral limb; and 2: contralateral limb weakness.

### 2.5. Assessment of Infarct Volume

The tMCAO rats treated with different administrations (*n* = 5) were euthanized, and their whole brains were rapidly removed 6 hours after reperfusion. Brains were dissected in coronal sections at 2 mm intervals. Brain sections were placed in a 1% 2,3,5-triphenyl tetrazole chloride (TTC) solution and incubated with 37°C for light avoidance for 10 min. The infarct area on either side of each section was evaluated using software [11].

### 2.6. Immunofluorescence Staining

Following a 30 min preservation at room temperature, the frozen rat cerebral sections were subjected to goat serum preincubation and subsequent overnight incubations using primary anti-NeuN (DF6145, AFFINITY) and anti-iNOS (AF0199, AFFINITY) under a 4°C condition, followed by a few rinses using PBS. Next, a further 1 h incubation proceeded using either FITC-labeled donkey anti-rat IgG secondary antibody or Texas Red-conjugated donkey anti-rabbit IgG secondary antibody. Finally, after a 5 min incubation of the sections using DAPI, a fluorescent microscope was utilized to accomplish the image acquisition.

### 2.7. Western Blot Assay

Extraction of proteins was accomplished from ischemic cerebral tissues, cells, and mitochondria, as per a formerly described protocol [13]. The proteins were then analyzed via western blot and shifted onto the PVDF (polyvinylidene fluoride) membranes (BioTrace; Pall Corp., USA). Incubation of the membranes proceeded using such primary antibodies followed by another 1 h incubation using secondary antibody (1 : 10000) at room temperature. For the photography and analysis of the target protein levels, an iBox Scientia 600 UVP imaging system (UVP, LLC, CA, USA) was utilized.

### 2.8. Transmission Electron Microscopy

After treatment, small pieces (1–3 mm cubes) of brain tissue were immersed in the fixative buffer for at least 2 h at 4°C [14]. The tissues were washed in 0.1 M cacodylate buffer and postfixed with 1% osmium tetroxide (OsO4)/1.5% potassium ferrocyanide (K2FeCN6) for 1 hour, washed in water three times, and incubated in 1% uranyl acetate in maleate buffer for 1 h followed by 3 washes in maleate buffer and subsequent dehydration in grades of alcohol (10 min each: 50%, 70%, and 90%; 2 × 10 min: 100%). Finally, brain tissues were embedded in TAAB 812 resin mixture and polymerized at 60°C for 48 h. In the case of mitochondrial pellets, mitochondria were fixed in 2.0% glutaraldehyde in 0.1 M sodium cacodylate buffer and pH 7.4 for 1 h at room temperature on a rocker. In the case of cells, isolation of mitochondria from BV2 microglial cells and SH-SY5Y cells was performed using the Cell Mitochondria Isolation Kit (Beyotime) according to the manufacturer's instructions [15]. Briefly, cells were incubated in mitochondrial lysis buffer for 10 min and homogenized using a tight pestle on ice. The homogenate was subjected to centrifuging at 600 g for 10 min. Then, the supernatant was collected and centrifuged again at 12,000 g for 10 min at 4°C to obtain pure mitochondria. All samples were double stained with uranium dioxide acetate and lead citrate. Finally, they were visualized by transmission electron microscopy (TEM).

### 2.9. MTT and TUNEL Assays

In order to trigger stroke-like neuronal impairment, this work conducted oxygen-glucose deprivation/reoxygenation (OGD/R) on SH-SY5Y cells through plating into the 6-well plates, which were processed with a 2 h oxygen-glucose deprivation and a 24 h reoxygenation. In order to obtain M1 microglial BV2 cells, OGD/R was used to stimulate microglial activation into stroke-like type M1 microglial BV2 cells in vitro. According to a previous report [16], the culture medium (CM) from M0 microglial BV2 cells (CM/M0-BV2) and M1 microglial BV2 cells (CM/M1-BV2) were removed, and the released mitochondria were collected from CM/M0-BV2 and CM/M1-BV2. After a 24 h coincubation of the released microglial mitochondria with the OGD/R-pretreated SH-SY5Y cells, a further 4 h incubation proceeded through addition of serum-free MTT-involving medium. As a last step, dimethyl sulfoxide was added to OGD/R-pretreated SH-SY5Y cells, followed by measurement of 490 nm optical density. This study evaluated cellular apoptosis through the terminal deoxynucleotidyl transferase-mediated dUTP nick end labeling (TUNEL) assay.

### 2.10. Isolation of Mitochondria

The microglial medium was first centrifuged at 1000 g for 10 mins to remove cell debris, the supernatant was retained, and the enrichment was collected as extracellular free mitochondria for 25 min at 13000 g [16].

### 2.11. ATP Measurement

A Luminescent Cellular Viability Assay Kit (Cat no #A6103S; Everbright, USA) was utilized for examining the relative levels of intracellular or extracellular ATP [15], which enabled cellular lysis and production of ATP-proportional luminescent signal. Initially, for determining intracellular ATP, cell lysate (50 *μ*l) was poured into 96-well microplates. Then, each wall was added with single-one-step reagent from the kit at an equivalent volume, followed by a 30 min incubation at room temperature. In the determination of extracellular mitochondrial ATP, cellular supernatant was subjected to a 10 min centrifugation at 1,000 g for the debris elimination. After a further 25 min centrifugation at 13,000 g, washing in PBS was (1 ml) proceeded, and then a serum- and phenol-free PBS or DMEM (50 *μ*l) was used to resuspend the pellet prior to a 30 min incubation at room temperature by adding single-one-step reagent from the kit at an equivalent volume. ATP content was measured using microplate reader.

### 2.12. Mitochondria Membrane Potential Measurement

For the mitochondrial health surveillance, the mitochondria membrane potential measurement was assessed with JC-1 dye (Cat no. #M8650; Solarbio). Initially, cellular supernatant was subjected to a 10 min centrifugation (1,000 g) for the debris elimination and then to a 30 min loading with JC1 dye (1 *μ*M) at 37°C. The JC1 dye enrichment in mitochondria was observed in a potential-dependent fashion, evident from shift of fluorescent emission from red (Ex 579 nm/Em 599 nm) to green (Ex 485 nm/Em 516 nm). A fluorescent microplate reader was utilized for assessing the mitochondrial membrane potential according to the fluorescent ratio.

### 2.13. Determination of ROS Production

To determine mitochondrial ROS production, extracellular mitochondria were treated with 5 *μ*M MitoSOX Red mitochondrial superoxide indicator (Invitrogen, Cat no #40778ES50) for 20 min at 37°C according to the manufacturers' protocols. Fluorescence was analyzed at excitation/emission maxima of 510/588 nm. To determine cellular ROS production, cells were incubated with 5 *μ*M CellROX Oxidative Stress Reagent (Invitrogen, Cat no #BB-47053) for 30 min long. The fluorescence was analyzed using microplate reader, at excitation/emission maxima of 485/520 nm for CellROX.

### 2.14. Statistical Analysis

All data were analyzed by an observer who was blind to the experimental protocol. Statistical calculations were performed with the GraphPad Prism software. Data were presented as mean ± SD. One-way ANOVA was employed for analyzing data statistically. Significant effects in analysis of variances were followed by Dunnett's multiple comparison post-hoc tests. Significance levels were set at ^∗^*P* < 0.05, ^∗∗^*P* < 0.01, and ^∗∗∗^*P* < 0.001.

## 3. Results

### 3.1. Activation of Microglia towards M1 Phenotype after IS

**Figure 2:**
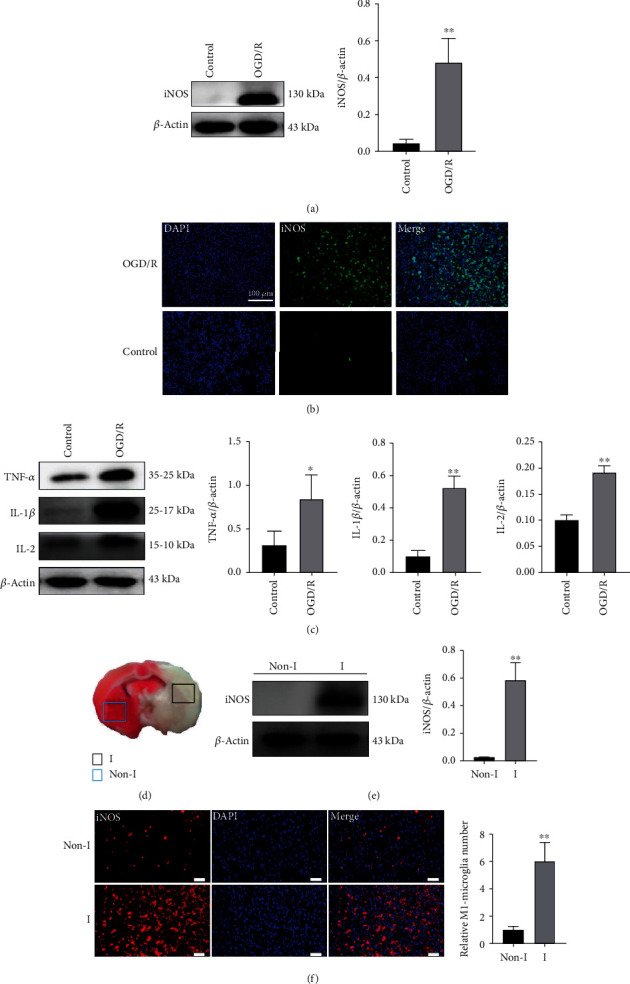
Microglia was activated to M1 type in vivo and in vitro after IS. Normal BV2 cells were regarded as the control group. (a) The activation of M1 type microglia was detected by determining the expression of iNOS (M1 type microglia marker) in OGD/R-stimulated BV2 cells (*n* = 3). Results are presented in a form of mean ± SD, and ^∗∗^ indicates *P* < 0.01. (b) Immunofluorescence image of expressing of iNOS in OGD/R-stimulated BV2 cells. Scale bar: 100 *μ*m. (c) IL-1*β*, IL-2, and TNF-*α* levels in OGD/R-stimulated BV2 cells based on western blotting (*n* = 3). Results are displayed in a form of mean ± SD; ^∗^*P* < 0.05 and ^∗∗^*P* < 0.01. (d) Schematic diagram showing brain slice as a comparison between infarct side and noninfarct side. (e) WB assay regarding iNOS protein level in infarct region and noninfarct region from brain tissues (*n* = 3). Results are displayed in a form of mean ± SD; ^∗∗^*P* < 0.01. (f) Immunofluorescence and quantitative assays of iNOS in infarct region and noninfarct region from brain tissues (*n* = 3). The relative number of M1 microglia was determined by calculating the ratio of the number of iNOS-positive cells in groups to the number of iNOS-positive cells in noninfarct region from brain tissues. Scale bar: 50 *μ*m. Results are displayed in a form of mean ± SD and ^∗∗^*P* < 0.01.

To investigate whether microglia were activated to M1 type after IS, a 6 h OGD was accomplished on microglia, followed by a 24 h reoxygenation. Western blot test and immunofluorescence results observed from Figures 2(a) and 2(b) revealed that the microglial M1 marker iNOS expression was increased after OGD/R, which proved that microglia were activated to M1 after OGD/R treatment in vitro. In addition, when microglia were collected after OGD/R, western blot results also showed that after being treated with OGD/R, inflammatory IL-1*β*, TNF-*α*, and interleukin-2 (IL-2) cytokine levels were increased (Figure 2(c)). To investigate the different degrees of activation of microglia on the ischemic and nonischemic sides, we sampled on the ischemic and nonischemic sides of tMCAO rats, as shown in Figure 2(d) (the white part represented the infarcted areas, whereas the red part represented the noninfarcted areas). We further confirmed microglial activation (M1) in ischemic region using immunofluorescence staining and western blotting. As shown in Figures 2(e) and 2(f), the stronger red fluorescence, representing iNOS, was observed in ischemic region compared with that in the nonischemic part. Meanwhile, the expression of iNOS in ischemic region was significantly increased. All these results indicated that tMCAO promoted the activation of microglia towards M1 type.

### 3.2. Dysfunction of Mitochondria in M1 Microglia by Enhancing Mitochondrial Fission and Producing Damaged Mitochondrial Fragmentation

**Figure 3:**
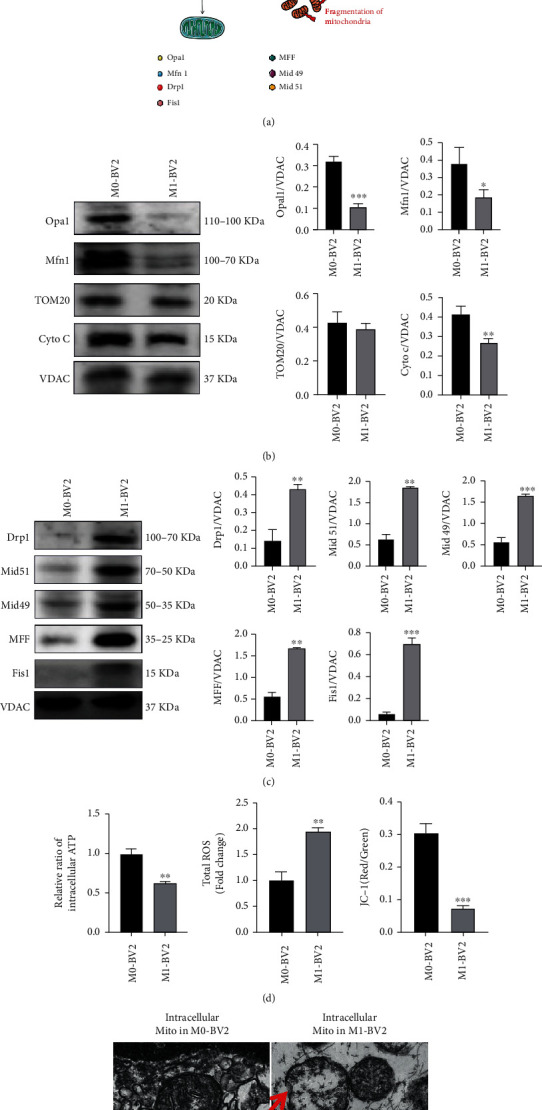
Mitochondrial changes in activated microglia (M1). (a) Schematic diagram of intracellular mitochondrial fusion and fission process in microglia after OGD/R. (b) Western blot assay of mitochondrial fusion protein (Opa1 and Mfn1), TOM20, and cytochrome c in mitochondria of M0 and M1 microglia (*n* = 3). Results are displayed in a form of mean ± SD; ^∗^*P* < 0.05, ^∗∗^*P* < 0.01, and ^∗∗∗^*P* < 0.001. (c) Western blotting findings of mitochondrial fission protein including MFF, Fis1, Mid49, and Mid51 in mitochondria of M0 and M1 microglia (*n* = 3). Data presented are mean ± SD; ^∗∗^*P* < 0.01 and ^∗∗∗^*P* < 0.001. (d) Mitochondrial function in M1 microglia and M0 microglia was investigated by determining ATP (*n* = 3), mitochondria membrane potential (*n* = 3), and ROS (*n* = 3). The relative ratio of intracellular ATP was determined by calculating the ratio of level of ATP in M0-BV2 and M1-BV2 cells to level of ATP in M0-BV2 cells. Results are displayed in a form of mean ± SD. ^∗∗^*P* < 0.01 and ^∗∗∗^*P* < 0.001. (e) Morphology of intracellular mitochondria in BV2 cells visualized under TEM, scale bar: 1.0 *μ*m.

In order to explore whether IS-mediated microglial M1 polarization had an impact on their mitochondrial function, we tested the changes in the expression of a series of related fission proteins and fusion proteins in mitochondria in both M0 and M1 type BV2 cells. As shown in the schematic diagram in Figure 3(a), under normal conditions, the processes of mitochondrial fusion and fission are a balanced state, but when IS occurs, microglia in the ischemic region is activated to M1 type. Aided by such fission proteins as Fis1 (mitochondrial adaptor fission 1), MFF (mitochondrial fission factor), and Mid49 and Mid51 (49- and 51 kDa mitochondrial dynamics proteins), Drp1 was recruited to the external mitochondrial membrane as a key mitochondrial regulatory protein, causing an exaggerated fission process. At the same time, the fusion proteins of mitochondria such as Opa1 (optic atrophy 1) and Mfn1 (mitofusin 1) were significantly downregulated. As a result, unhealthy mitochondria are produced, and function of mitochondria is severely disrupted in M1 microglia. Therefore, we performed western blot for detecting fission proteins and fusion proteins, and VDAC in mitochondria extracts from the corresponding group was used as a loading control according the previous report [17]. Compared with resting microglia (M0), the expressions of mitochondrial fission protein such as MFF, Fis1, Mid49, and Mid51 were increased (Figure 3(c)), and fusion protein levels such as Opa1 and Mfn1 were decreased in M1 microglia (Figure 3(b)). Furthermore, the mitochondrial function of M1 microglia was seriously damaged by reducing the mitochondrial membrane potential, inhibiting production of intracellular ATP, and increasing the levels of intracellular ROS (Figure 3(d)).

In order to more directly observe the structural changes of mitochondria in microglia after OGD/R, TEM was applied to observe the morphology of mitochondria. As shown in Figure 3(e), TEM results showed that compared with mitochondria in M0 microglia, mitochondria in M1 microglia swelled up, and the cristae of mitochondria partially disintegrated and became transparent density. All results demonstrated that when stroke occurred, the mitochondrial structure in activated microglia (M1) changed, and the mitochondrial function was interfered.

### 3.3. The Release of Damaged and Unhealthy Mitochondrial Fragmentation from Activated Microglia (M1)

**Figure 4:**
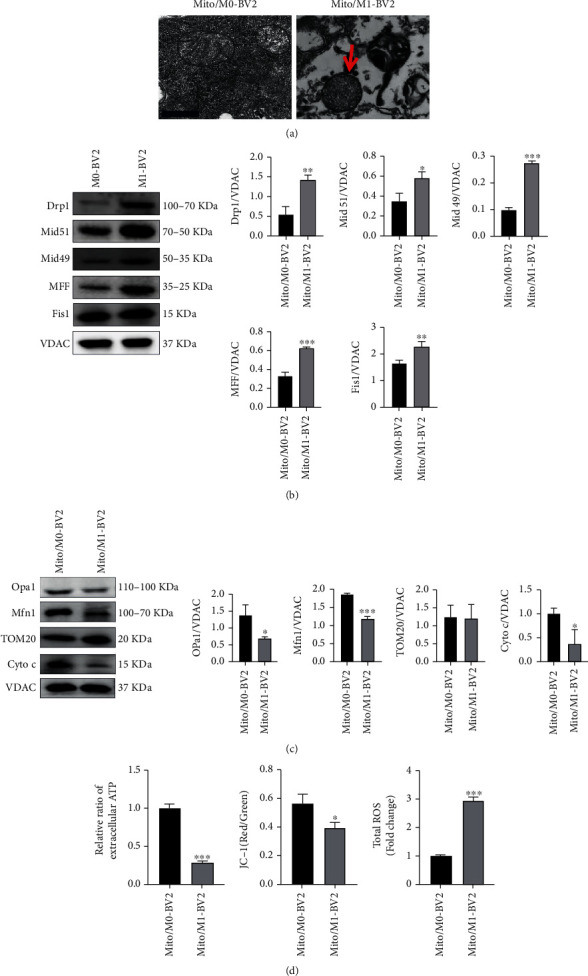
Functional changes of released mitochondria secreted by activated microglia (M1). (a) Morphological characterization of released mitochondria isolated from culture medium of M0 microglia (Mito/M0-BV2) and M1 microglia (Mito/M1-BV2). Scale bar: 1.0 *μ*m. (b) Levels of proteins associated with mitochondrial fission in Mito/M0-BV2 and Mito/M1-BV2 based on the western blotting (*n* = 3). Results are displayed in a form of mean ± SD; ^∗^*P* < 0.05, ^∗∗^*P* < 0.01, and ^∗∗∗^*P* < 0.001. (c) The expression of associated fusion protein, TOM20, and Cyto C in Mito/M0-BV2 and Mito/M1-BV2 (*n* = 3). Results are displayed in a form of mean ± SD, ^∗^*P* < 0.05, and ^∗∗∗^*P* < 0.001. (d) After OGD/R for 24 hours, function of Mito/M0-BV2 and Mito/M1-BV2 was detected by checking ATP (*n* = 3), mitochondria membrane potential (*n* = 3), and ROS (*n* = 3). The relative ratio of extracellular ATP in secreted mitochondria from BV2 cells was determined by calculating the ratio of level of ATP in Mito/M0-BV2 and Mito/M1-BV2 to level of ATP in Mito/M0-BV2. Results are displayed in a form of mean ± SD; ^∗^*P* < 0.05 and ^∗∗∗^*P* < 0.001.

In recent years, studies have shown that cells can secrete mitochondria for regulating mitochondrial activity and biological function in nearby cells [18]. As shown in Figure 4(a), the results of TEM not only proved that microglia can secrete mitochondria out of the cell but also justified that the morphology of mitochondria secreted by M1 microglia represented by Mito/M1-BV2 had been changed as evidenced by incomplete and fuzzy membrane, vacuolization, and fragment of cristae as compared to mitochondria secreted from M0 microglia represented by Mito/M0-BV2. At the same time, the mitochondrial-related proteins in released mitochondria were detected by western blot. As was clear from Figures 4(b) and 4(c), the fission protein in released mitochondria of M1 microglia was increased, and the fusion protein was decreased, proving that secreted mitochondria by M1 microglia were damaged and fragmented. In order to directly observe the changes of mitochondrial function, ATP, membrane potential, and ROS of released mitochondria were detected. As shown in Figure 4(d), mitochondrial ATP in released mitochondria secreted by activated microglia (M1) was significantly decreased, membrane potential was reduced, and ROS was massively produced. These results revealed that mitochondria can also be secreted out of the cell after microglia was activated to M1 type, and the function of released mitochondria was also damaged.

### 3.4. M1 Microglia Released Mitochondria Can Transfer to Neuronal Cells and Affect the Function of Neuronal Mitochondria

**Figure 5:**
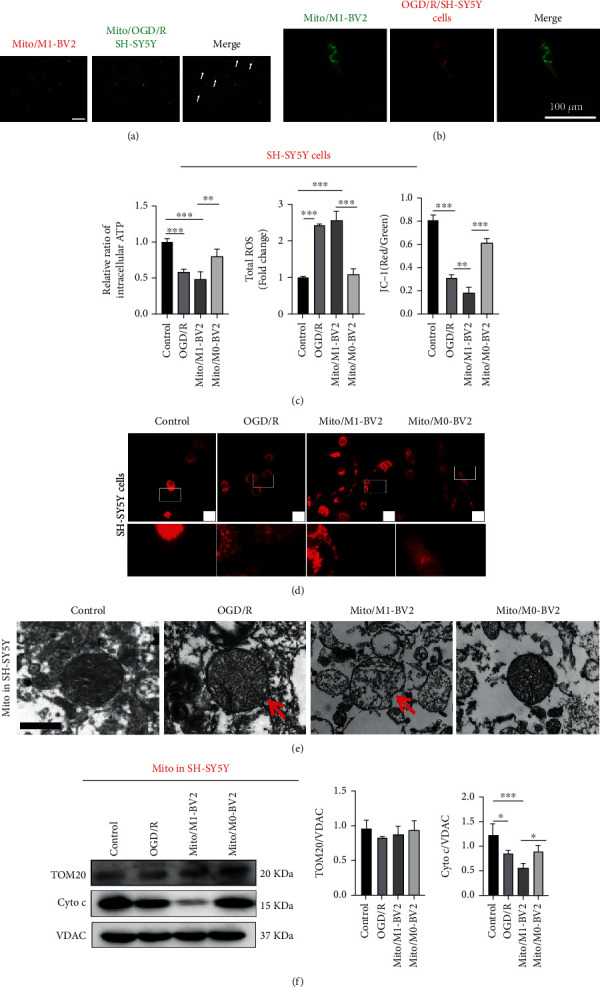
Microglial mitochondria can transfer to neurons and fuse with neuronal mitochondria for affecting neuronal mitochondrial function. (a) Mitochondria were obtained from the culture medium of M1 BV2 cells (Mito/M1-BV2) and OGD/R-stimulated SH-SY5Y cells (Mito/OGD/R/SH-SY5Y). Confocal image of fusion of Mito/M1-BV2 (red) and Mito/OGD/R/SH-SY5Y (green). Scale bar: 100 *μ*m. (b) Confocal image of fusion of Mito/M1-BV2 (green) and intracellular mitochondria in SH-SY5Y cells (Mito in SH-SY5Y) (red). Scale bar: 100 *μ*m. (c) Detection of mitochondrial ATP (*n* = 3), JC-1(*n* = 3), and ROS (*n* = 3) in intracellular mitochondria of OGD/R-stimulated SH-SY5Y cells incubated with Mito/M1-BV2 and Mito/M0-BV2 for 24 hours. Results are displayed in a form of mean ± SD. ^∗∗^*P* < 0.01 and ^∗∗∗^*P* < 0.001. (d) Confocal images of Mito Tracker Red-labeled intracellular mitochondria of OGD/R-stimulated SH-SY5Y cells (Mito in SH-SY5Y) treated with Mito/M1-BV2 and Mito/M0-BV2 at a scale of 100 *μ*m. (e) TEM of mitochondrial structure in intracellular mitochondria of OGD/R-stimulated SH-SY5Y cells (Mito in SH-SY5Y) treated with Mito/M1-BV2 and Mito/M0-BV2. Scale bar: 1.0 *μ*m. (f) Western blot analysis of TOM 20 and cytochrome c in intracellular mitochondria of OGD/R-stimulated SH-SY5Y cells (Mito in SH-SY5Y) treated with Mito/M1-BV2 and Mito/M0-BV2 (*n* = 3). Results are displayed in a form of mean ± SD; ^∗^*P* < 0.05 and ^∗∗∗^*P* < 0.001.

It is well known that mitochondrial transfer can initiate cell reprogramming and affect the function of mitochondria in recipient cells, thus saving cell activity or aggravating cell death [19]. In order to prove mitochondrial crosstalk between microglia and neurons, mitochondria were obtained from the culture medium of M1-BV2 cells (Mito/M1-BV2) and OGD/R-stimulated SH-SY5Y cells (Mito/OGD/R/SH-SY5Y). Mito/M1-BV2 was stained with MitoTracker Red and coincubated with Mito/OGD/R/SH-SY5Y labeled with MitoTracker Green for 60 mins. As shown in Figure 5(a), there was obvious colocation between the green fluorescence and red fluorescence, indicating that mitochondrial fusion occurred between released mitochondria which were isolated from culture medium of microglia and neuronal mitochondriam in vitro. In order to further prove that released microglial mitochondria can enter neurons and fuse with mitochondria in neurons after entering neuronal cells, Mito/M1-BV2 was stained with MitoTracker Green and coincubated with SH-SY5Y cells in which intracellular mitochondria in SH-SY5Y cells (Mito in SH-SY5Y) were labeled with Mito Tracker Red. As shown in Figure 5(b), green fluorescence emitted by Mito/M1-BV2 merged well with red fluorescence, a marker of intracellular mitochondria in SH-SY5Y cells (Mito in SH-SY5Y), indicating obvious transfer and fusion occured between released microglial mitochondria and neuronal mitochondria. In order to confirm the effects of Mito/M1-BV2 on mitochondrial function of neurons, we measured the ATP, JC-1, and ROS of OGD/R-stimulated SH-SY5Y cells treated with released microglial mitochondria. According to Figure 5(c), following a 24 h coincubation of OGD/R-stimulated SH-SY5Y cells with Mito/M1-BV2, the ATP and membrane potential of mitochondria were significantly decreased, and the generation ROS was increased in OGD/R-stimulated SH-SY5Y cells. The fluorescence image (Figure 5(d)) showed that the intracellular mitochondria in OGD/R-stimulated SH-SY5Y cells treated with Mito/M1-BV2 showed vacuolar structure and became dot circular and shorter in length. In order to more directly explain the released M1 microglial mitochondria-mediated changes of neuronal mitochondria, the morphology of mitochondria in neurons was also observed by TEM. As shown in Figure 5(e), after Mito/M1-BV2 was incubated with OGD/R-stimulated SH-SY5Y cells, the mitochondria in OGD/R-stimulated SH-SY5Y cells showed vacuolization, the mitochondrial cristae partly disintegrated, and even the structure and membrane of the mitochondria were disrupted by the magnanimous release of cytochrome c (Figure 5(f)).

### 3.5. Released Mitochondria from M1 Microglia Can Effectively Aggravate the Damage of OGD/R-Stimulated Neurons

**Figure 6:**
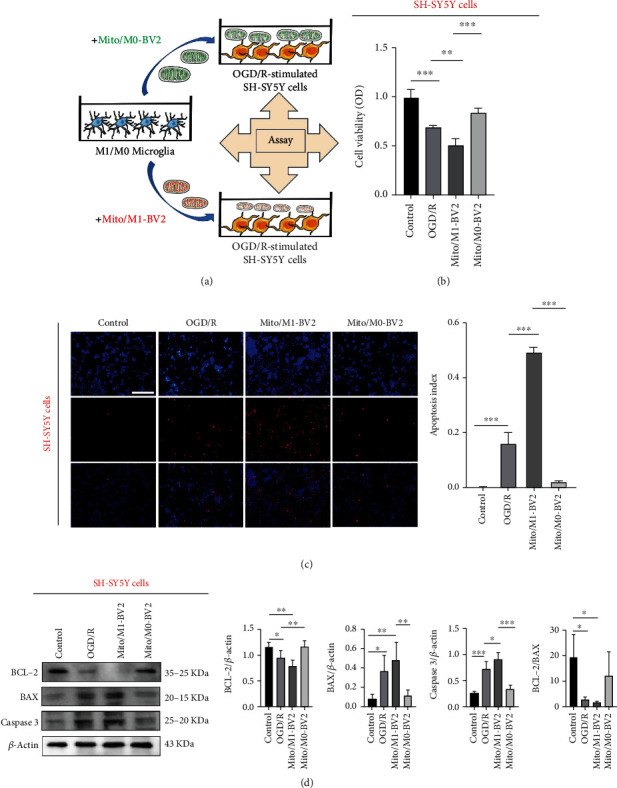
Released mitochondria secreted by activated microglia (M1) can aggravate the damage of OGD/R-stimulated neurons. (a) Experimental chart. (b) Cell viability after coincubation of OGD/R-stimulated SH-SY5Y cells with Mito/M1-BV2 and Mito/M0-BV2 for 24 hours (*n* = 3). Results are displayed in a form of mean ± SD; ^∗∗^*P* < 0.01 and ^∗∗∗^*P* < 0.001. (c) Imaging observation and quantitative findings of apoptotic cells after coincubation of OGD/R-stimulated SH-SY5Y cells treated with Mito/M1-BV2 and Mito/M0-BV2 for 24 hours (*n* = 3). Apoptosis Index was determined by calculating the ratio of number of TUNEL positive cells to total Number of nuclei. Scale bar: 100 *μ*m. Results are displayed in a form of mean ± SD; ^∗∗∗^*P* < 0.001. (d) Apoptotic protein levels, including BAX, BCL-2, and caspase 3 in OGD/R-stimulated SH-SY5Y cells treated with Mito/M1-BV2 and Mito/M0-BV2 based on the western blotting (*n* = 3). Results are displayed in a form of mean ± SD. ^∗^*P* < 0.05, ^∗∗^*P* < 0.01, and ^∗∗∗^*P* < 0.001.

Basing on our previous experimental results, we concluded that released mitochondria from microglia can enter into neurons and fuse with neuronal mitochondria, affecting the function of mitochondria in neurons. Therefore, we further explored whether released mitochondria from M1 microglia affected the mitochondria-mediated apoptosis of neurons. The released mitochondria in culture medium of BV2 cells were obtained and incubated with OGD/R-stimulated SH-SY5Y cells at 37°C for 24 hours (Figure 6(a)). We used the MTT method to examine the effect of mitochondria from microglia on regulating neuronal cell viability. The results shown in Figure 6(b) demonstrated that compared with untreated OGD/R-stimulated SH-SY5Y cells, addition of Mito/M1-BV2 cells significantly reduced the viability of neurons at 51.53% and triggered severe cell apoptosis effects as evidenced by higher number of TUNEL-positive cells (Figure 6(c)), confirming that released mitochondria from M1 microglia significantly exacerbated the injury of OGD/R-stimulated neurons. In addition, we also explored the mechanism of apoptosis of OGD/R-stimulated neurons induced by microglia released mitochondria. The data (Figure 6(d)) showed that after treated with Mito/M1-BV2, the downregulated expression of BCL-2, a protein associated with antiapoptosis, was noted, while the expression of BAX, a protein associated with apoptosis, was upregulated in OGD/R-stimulated neurons. Then, in response to cell stimulation, more cytochrome c was finally released from mitochondria through pores and other channels and then activated caspase-3 and downstream caspase cascade to induce cell death. Taken together, it was proved that the released mitochondria secreted by activated microglia (M1) were transferred to neurons and contributed to accelerating the progression of ischemic damage via activating mitochondria-mediated apoptosis.

### 3.6. M1 Microglia Released Unhealthy Mitochondria Exacerbated Neurological Deficit in tMCAO Rats In Vivo

**Figure 7:**
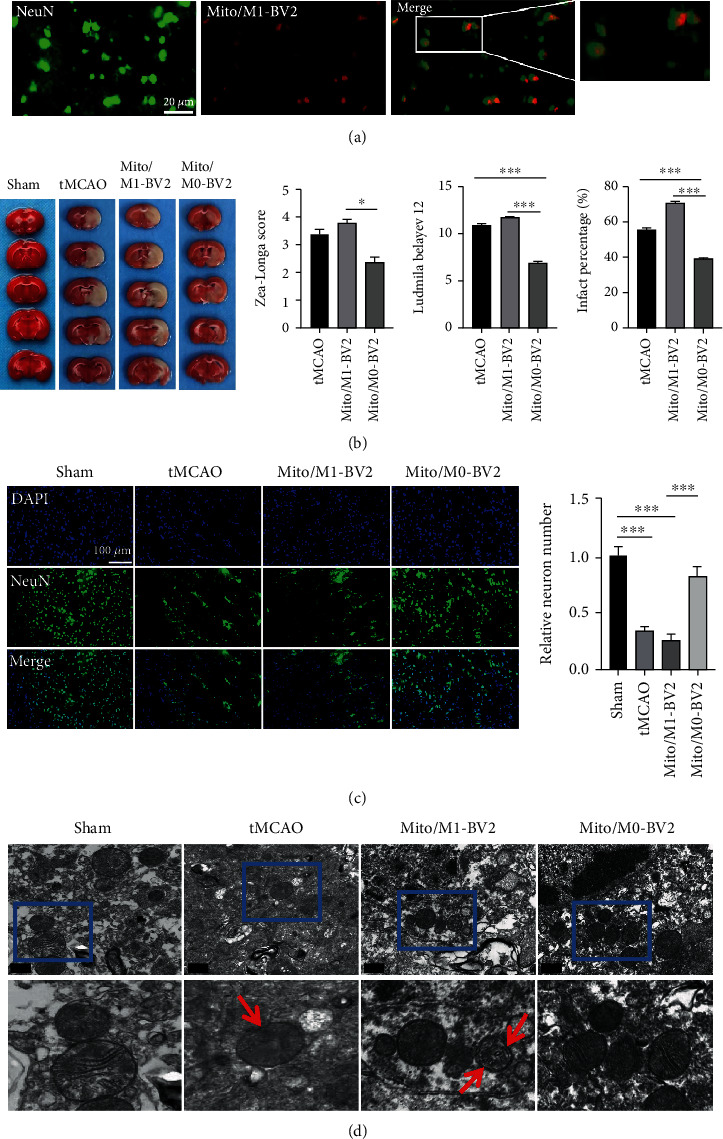
Neurological effects of Mito/M1-BV2 and Mito/M0-BV2 on tMCAO rats. (a) After Mito/M1-BV2 was labeled red and injected into the ischemic cortex of rats, the immunofluorescent staining revealed colocalization of Mito Tracker Red-labeled microglial mitochondria with neurons labeled with anti-NeuN (green). Scale bar: 20 *μ*m. (b) Cerebral sections (*n* = 5) were stained using 2,3,5-triphenyltetrachloroammonium (TTC). After ischemia for 2 h and reperfusion for 6 h, the infarct volume was calculated. In addition, Ludmila belayev (*n* = 5) and Zea-longa neurological scores (*n* = 5) were determined. The data indicate mean ± SD, ^∗^*P* < 0.05, and ^∗∗∗^*P* < 0.001. (c) The immunofluorescent staining of NeuN-positive cells (green) in ischemic region of tMCAO rats treated with Mito/M0-BV2 and Mito/M1-BV2. NeuN antibody was utilized for neuron staining in ischemic cerebral tissues of tMCAO rats (*n* = 3). DAPI (blue) served as the nuclear marker. NeuN-positive neuronal counts were quantified. The relative neuron number was determined by calculating the ratio of the number of NeuN-positive cells in groups to the number of NeuN-positive cells in the sham group. Scale bar: 100 *μ*m. Data presented are mean ± SD. ^∗∗∗^*P* < 0.001. (d) TEM images of mitochondria in ischemic cerebral tissues of tMCAO rats. Scale bar: 0.5 *μ*m.

As we have proved that activated microglia (M1) released unhealthy mitochondria can significantly regulate neuronal apoptosis and interrupt mitochondrial function of neurons in vitro, we further clarified whether activated microglia (M1)-mediated transfer of unhealthy mitochondria into neurons aggravated damage of ischemic stroke in a tMCAO model. First, released mitochondrial particles from activated M1 microglia were collected and labeled with Mito Tracker Red. Then, they were injected directly into the cortex around the infarction in tMCAO rats. After injection into the ischemic cortex of rats for 6 hours, immunostaining results (Figure 7(a)) showed that the transplanted mitochondria of microglia did exist in neurons, which meant that exogenous microglia also had mitochondrial crosstalk with neurons in vivo. To investigate how the microglia released mitochondria affected neurological regulation, rats were randomly assigned to four groups (*n* = 5 per group): sham group without any treatment (Sham), tMCAO group with PBS treatment (tMCAO), tMCAO treated with Mito/M1-BV2, and tMCAO treated Mito/M0-BV2. After 2 h occlusion, the suture was removed, and PBS, Mito/M1-BV2, and Mito/M0-BV2 were directly injected into peri-infarct cortex of tMCAO rats. Then, TTC staining was performed at 6 h of treatment. As shown in Figure 7(b), an elevated infarct area ratio was noted in the Mito/M1-BV2-treated tMCAO group as compared to the PBS-treated tMCAO group, while the Zea-Longa and Ludmila Belayev neurological scores in the Mito/M1-BV2-treated tMCAO group increased significantly, indicating that extracellular mitochondria released from M1 microglia exacerbated neurological dysfunction after entering tMCAO rats. As the damage or loss of neurons will affect the recovery of neuronal function, the frozen sections of rat brain were stained by the immunofluorescence method. The neurons were labeled with NeuN, and the number of neurons was observed and calculated. The quantitative analysis results (Figure 7(c)) showed that Mito/M1-BV2 did further induce neuronal injury by showing less NeuN-positive cell counts in the ischemic penumbra when compared to that in the PBS-treated tMCAO group. In order to explore whether the implantation of Mito/M1-BV2 would affect the mitochondrial function in the tMCAO rat brain, the in vivo mitochondrial morphology was examined under TEM. As shown in Figure 7(d), compared with the sham group, the damaged mitochondrial structure, fracture, and disappearance of the mitochondrial cristae were observed in ischemia brain tissue of tMCAO rats treated with Mito/M1-BV2. The above results showed that the released mitochondria by activated M1 microglia exerted a detrimental effect on ischemic brain by interfering with the morphology and function of mitochondria in vivo.

## 4. Discussion

Neurons and glial cells maintain homeostasis in the central nervous system (CNS) through different intracellular and intercellular signaling mechanisms [20]. Some soluble factors were exchanged between cells through direct intercellular contact, so as to realize the short-distance and long-distance transfer of substances between cells [21]. In recent years, the transcellular transfer of mitochondria has become an important representative of this communication. The crosstalk mechanism of mitochondria between cells provides potential hints for regulating neuronal survival. Our previous research also confirmed that after stroke, injured mitochondria from activated type A1 astrocytes can lead to the aggravation of neuronal damage and prolonged recovery after ischemia stroke [22].

Microglia has also been verified to play a vital role in the process of brain recovery in diseases such as Alzheimer's disease and Hodgkin's disease [23]. When the disease occurs, a series type of activated microglia demonstrated different mitochondrial function [24]. However, during the period of stroke, the crucial effects of activated microglia released mitochondria on regulating pathological formation and development of disease are still unclear. In this study, a significant discovery revealed that when stroke occurred, activated microglia (M1) exhibited injured mitochondrial status, such as damaged mitochondrial function and activity. The level of mitochondrial ATP in M1 microglia decreased, the membrane potential was reduced, and the oxidative stress was induced. At the same time, unhealthy mitochondria were released from M1 microglia and subsequently transferred to neurons through the mitochondrial transmission mechanism between cells, thus reducing the supply of ATP in neurons, resulting in the loss of neuronal mitochondrial function and aggravation of damage.

More and more studies have emphasized the important role of microglia in phenotypic transformation [25]. Some people believe that this switch from M0 microglia to M1 microglia after stroke triggers the strong inflammatory response which is detrimental to repair tissue damage [26]. At present, some preclinical trials have used new drugs, including antioxidants or neuroprotective agent, to reduce stroke-induced neuroinflammation [27]. However, there are few effective treatments to quickly reverse mitochondrial dysfunction. In recent years, the term “mitochondrial transmission” has opened up a new vision for many central nervous system diseases, such as neurodegenerative diseases and spinal cord injury [28]. The mitochondrial function of damaged cells can be regulated by endogenous or exogenous mitochondrial transfer, which has been confirmed in Parkinson's disease, spinal cord injury, and stroke models [29]. Previous studies showed that after cerebral ischemia-reperfusion injury, injecting exogenous mitochondria directly into the lateral ventricle can regulate cell oxidative stress and apoptosis, even participating in neuroprotection [30]. Our study found that apart from the M1 microglia mediated the release of inflammatory factors after ischemic stroke, a great amount of damaged mitochondrial fragmentation was produced in M1 microglia by upregulating binding to the Drp1 to other related mitotic protein receptors. Finally, mitochondria secreted by activated microglia (M1) can exacerbate ischemic stroke injury through their transition to injured neurons and subsequently fusion with mitochondria in neurons. As a result, exogenous mitochondria released by M1 microglia aggravated neuronal mitochondrial damage and interfered neuronal mitochondrial function. In vivo, transplantation of M1 microglia released mitochondria significantly aggravated ischemic damage in tMCAO rats by enhancing the infarct percentage and lowering neurological performance. We demonstrated the key role of mitochondria in stroke after microglia polarization in vivo and in vitro.

Mitochondrial transfer provides a new insight for the survival and regeneration of neurons after stroke. Our preliminary research also suggested that M0 microglia released mitochondria seemed to have a significant therapeutic effect on ischemic stroke. Treatment of M0 microglia released mitochondria significantly increased the cell viability of OGD/R-stimulated neurons (Figure 6(b)) and mitigated ischemic damage in tMCAO rats by reducing the infarct area and enhancing neurological performance (Figures 7(b) and 7(c)). All data revealed that M0 microglia released healthy mitochondria contributed to neuroprotection. However, before mitochondrial transfer is clinically utilized, a better understanding of the mechanisms of mitochondrial transmission and the potential adverse effects is needed to further investigate.

## 5. Conclusion

After ischemic stroke occurred, microglia was activated into M1 type and released damaged mitochondria as evidenced by decreasing mitochondrial membrane potential, reducing ATP production and increasing ROS generation. Subsequently, these released damaged microglial mitochondria were transmitted to injured neurons and fused with mitochondria in neurons for aggravating mitochondria-mediated apoptosis. This study indicated that M1 microglia-mediated mitochondrial transfer into neurons may provide a potential pathological mechanism on regulating stroke-related brain dysfunction.

## Data Availability

The data used to support the findings of this study are available from the corresponding authors upon request.
